# Lifestyle and silymarin: a fight against liver damage in NAFLD associated - prediabetic disease

**DOI:** 10.1007/s40200-020-00576-3

**Published:** 2020-06-30

**Authors:** Cosimo Colletta, Alessandro Colletta, Giuseppe Placentino

**Affiliations:** 1 Division of Internal Medicine, Hepatology COQ, Madonna del Popolo Hospital, via Lungolago Buozzi 25, 28887 Omegna, VB Italy; 2 Department of Medicine, Boston Medical Center, Boston, MA USA; 3 Diabetes Clinic, Castelli Hospital ASL VCO, Verbania, Italy

**Keywords:** Non-alcoholic fatty liver disease, Prediabetes, Body mass index, PNPLA3, Silymarin

## Abstract

**Background:**

Nonalcoholic fatty liver disease (NAFLD) is common in both prediabetic patients and healthy overweight individuals, yet it remains understudied. This study investigates the effects of hepatic steatosis on fibrosis and evaluates the major predictors of liver injury in prediabetes and whether this damage is reversible with Mediterranean diet and administration of the nutraceutical silymarin.

**Methods:**

First, a case-control study was conducted in which 212 patients with prediabetes, not known to have NAFLD, and 126 healthy controls underwent clinical evaluation, transient elastography with measurement of liver stiffness (LS) and controlled attenuation parameter (CAP). Subsequently, the 212 prediabetic patients were enrolled into a prospective randomized interventional study: 104 were allocated to Mediterranean diet alone while 108 followed Mediterranean diet plus supplementation with silymarin (a flavonolignan complex isolated from *Silybum marianum* and *Morus alba*). The administered silymarin dose was 210 mg twice daily for 6 months. Clinical and instrumental evaluations were repeated at the end of the 6 month-study period. Prediabetics were genotyped for patatin like phospholipase domain containing 3 (PNPLA3).

**Results:**

In the case-control study, 29% of prediabetic patients have significant fibrosis defined as LS ≥ 7.9 kPa vs only 3% of controls (*p* < 0.001). PNPLA3 genotype CG/GG are significantly associated with significant fibrosis LS ≥ 7.9 relative to CC genotype χ2(1) = 76.466, *p* < 0.001. Binomial regression analysis shows that increase in BMI, ALT and AST are significantly associated with increased likelihood of significant fibrosis (χ2(7) = 191.9, *p* < .001) prior to intervention. In the randomized interventional study, prediabetics following Mediterranean diet alone (group 1) experienced a significant regression of fibrosis and decrease in ALT, HbA1c, FBS after 6 months (*p* < 0.001); similar findings were observed in patients following Mediterranean diet plus silymarin regimen (group 2); group 2 had a significant decrease in HbA1c relative to group 1 (95% CI: 37.8–38.6 vs 39.5–40.3, *p* < 0.001).

**Conclusion:**

PNPLA3 genotype CG/GG and elevated BMI are the major predictors of significant fibrosis in prediabetic patients prior to intervention in this study. Mediterranean diet either alone or with silymarin treatment for 6 months leads to significant regression of liver damage and improvement of the glycemic profile in prediabetic patients. Yet, as combination treatment of silymarin with Mediterranean diet shows significant reduction of HbA1c when compared to diet alone, this suggests that silymarin may exert an independent anti-glycemic action.

## Introduction

NAFLD may present as a spectrum of disease from asymptomatic steatosis with or without elevated aminotransferases to cirrhosis with complications of liver failure and hepatocellular carcinoma [1].

The interaction between genetic background and environmental factors plays an important component in the pathogenesis of NAFLD [2]. With regard to the genetic factors involved, a single-nucleotide polymorphism occurring in the sequence of the human patatin-like phospholipase domain-containing 3 gene (PNPLA3), known as I148M variant, is one of the most investigated variants as it is associated with increased of developing hepatic steatosis and progressing to advanced fibrosis [3].

Specifically, the rs738409 C > G variant of PNPLA3 gene and its encoded protein adiponutrin have been associated with a variety of roles, ranging from an acquired lipogenic function to a loss of lipolytic activities [4]. NAFLD is closely associated with obesity, and due to the increased prevalence of metabolic syndrome among young age groups, NAFLD is to affect a substantial proportion of our population [5].

The high prevalence of NAFLD and its low risk of progression to steatohepatitis make a large-scale screening strategy for NAFLD unfitting for the general population [6]. Thus, focusing our screening on individuals at higher risk of progressing to more advanced liver disease is more cost-effective. Patients with prediabetes fall in this category as they are not only at high risk of developing NAFLD [7], but also show a higher rate of progression to significant hepatic fibrosis relative to other patients with NAFLD [8]. Furthermore, as the prevalence of NAFLD in prediabetic patients remains uncertain and effective screening strategies in this patient population are lacking, we observe a higher frequency of non-referrals, especially when transaminases are deceivingly within a normal range.

The way of managing patients with suspected liver disease has been recently modified by the advent of transient elastography (TE) [9]. This methodology accurately quantifies liver fat by measuring the so-called controlled attenuation parameter (CAP), which identifies steatosis independently from the presence of fibrosis [10]. Thus, the use of TE with CAP measurement in prediabetes may allow us to risk stratify these patients, to assess their progression of NAFLD and to evaluate the possible reversibility of their liver disease in the context of specific intervention strategies.

Lifestyle modifications such as dietary changes and increase in physical activity have been previously documented as interventions for control of NAFLD [11, 12]. Exercise contributes to NASH regression by reducing insulin resistance, decreasing the synthesis of free fatty acids (FFAs) and their storage into triglycerides [12]. Insulin sensitizers like metformin have shown improvement of liver enzymatic factors but without any effect of liver histology [13]. Similarly, therapy with herbal drugs like silymarin, a complex of flowers and leaves of *Silybum marianum* (milk thistle), has shown improvement of transaminases in the context of liver disease [14, 15]. Nevertheless, the combined effect of nutraceuticals and diet on liver fibrosis and its possible regression in prediabetic patients remains understudied.

With these considerations in mind, we herein propose to investigate which specific genetic factors and patients’ demographics can be effectively utilized as critical variables in the risk stratification analysis of prediabetic patients with NAFLD. Finally, by using TE with CAP measurement, we specifically evaluate whether prediabetic patients who undergo lifestyle changes and use of silymarin have a significant regression of fibrosis relative to their initial baseline.

## Methods

### Patients

First, a case-control study was conducted; in this initial phase, we have recruited and enrolled 212 patients in prediabetic state (HbA1c 5.7–6.4% or 39–46 mmol/mol) who had not yet received pharmacological treatment and 126 healthy controls, all with BMI ranging from 25 to 29. All patients underwent clinical assessment, transient elastography with measure of liver stiffness (LS) and controlled attenuation parameter (CAP).

At the time of enrolment, a sample of blood was taken for biochemical evaluations and patients were registered for age, sex and BMI. The inclusion criteria were as follows: age ≥ 18 years and prediabetic subjects who had never been treated for more than 12 weeks with antidiabetic drugs or with diet and exercise.

The exclusion criteria were as follows: positive serology for hepatitis C virus (HCV) or hepatitis B virus (HBV); concomitance of other causes of chronic liver disease (autoimmune hepatitis, hemochromatosis, cholestatic liver disease, drug-induced damage); presence of focal hepatic lesions of suspected malignant origin; excessive consumption of alcoholic beverages (≥3 drinks per day); obesity (BMI ≥ 30 kg/m^2^); inability to obtain valid TE measurements.

Total cholesterol and HDL; LDL cholesterol and triglycerides were measured by enzymatic methods. Glycosylated haemoglobin (HbA1c) was analysed by high pressure chromatography. Plasma glucose concentration was measured by hexokinase (ADVIA, Siemens Healthcare, Leverkusen, Germany). Aspartate aminotransferase (AST), alanine aminotransferase (ALT) and gamma-glutamyl transferase (GGT) were measured by enzymatic methods (Advia 1800 Chemistry System, Siemens Healthcare Diagnostics, Leverkusen, Germany).

### Randomized intervention study

After the case-control study phase, pre-diabetic patients were asked to participate in this open label, prospective randomized interventional study in accordance with the ethical guidelines of the Helsinki Declaration of 1975 and after approval by the local Ethics Committee (IRB n. 176/18). All consecutive adult patients with prediabetic state (HbA1c from 5.7 to 6.4%), not under pharmacological treatment, evaluated at the Diabetes Clinic of the Castelli Hospital in Verbania and at the Hepatology Clinic of the Madonna del Popolo Hospital in Omegna, in Northern Italy, between March 2017 and March 2018, were offered to participate to this study, according to the protocol approved. A written informed consent was obtained from all eligible patients (*n* = 212, of whom *n* = 157 were males) before participation. All were subjected to full history and clinical examination to assess history of hypertension, history of drug use, any chronic illness, and family history of diabetes.

All patients met, at least once, with a dietician for nutritional guidance, and were encouraged to start and maintain a low-calorie diet before study enrollment. The moderate-fat, restricted-calorie, Mediterranean diet was rich in vegetables and low in red meat, with poultry and fish replacing beef and lamb. We restricted energy intake to 1500 kcal per day for women and 1800 kcal per day for men.

At enrollment, 210 mg BID of silymarin (a flavonolignan complex isolated from *Silybum marianum* and *Morus alba* [Meda Pharma]) were added to the regimen of 108 patients (group 2) and supplementation was continued for the entirety of the study (6 months). At each visit, we enquired from the participants about any adverse events and medication compliance.

### NAFLD evaluation

Abdominal ultrasonography was performed after a fasting period of 12 h, by a radiologist who was blinded about the patients’ information. All patients underwent ultrasound examination of the liver; a sagittal sonographic plane of the section demonstrating the hepatic parenchyma and right kidney echogenicity was used for determination of liver parenchyma echogenicity [16]. With the same kidney cortex and liver parenchyma echogenicity it is evaluated as normal, no fatty liver (Grade 0). The liver steatosis was classified into 3 grades, as follows: Mild (Grade 1) - minimal diffuse increase in hepatic echogenicity, normal visualization of the diaphragm and intrahepatic vessels; Moderate (Grade 2) - medium diffuse increase in hepatic echogenicity, impaired visualization of intrahepatic vessels and diaphragm; Severe (Grade 3) - severe diffuse increase in hepatic echogenicity. Posterior segment of the right hepatic lobe is difficult to display. Failure to visualize the walls of the intrahepatic vessels and a marked decrease in the reflectivity of the hemidiaphragm.

LS was evaluated by TE (FibroScan®, Echosens, Paris, France). Measurements of LS and CAP were performed on the same day as abdominal ultrasonography after fasting for at least 12 h. The LS measurements from TE were performed on the right lobe of the liver through the intercostal space of patients in the position of dorsal decubitus with the right arm in maximum abduction.

TE was performed by a blind expert technician on patient clinical data. The median value of the successful measurements was selected as representative of the LS and CA*P* values ​​of the subjects. The CAP measured the ultrasonic attenuation at 3.5 MHz using the signals acquired by TE [17]. As an indicator of variability, the IQR ratios of the LS and CAP values ​​with respect to the median values ​​(IQR/M and IQR/MCAP, respectively) were calculated. In this study, only procedures with at least ten valid measurements, a success rate of at least 60% and an IQR/M value of LS less than 0.3 were considered reliable and used for the statistical analysis.

To correct variability in the general population, LS values ​​in prediabetic patients were compared to those observed in 126 healthy blood donors, (*n* = 94 males, 75%; *p* = 0.861). To define the presence of significant fibrosis, we used the cut-off value of 7.9 kPa, as proposed by others [18]. Furthermore, in prediabetics we measured the CAP, to classify hepatic steatosis by measuring the degree of US attenuation by liver fat [19]. CAP values ​​range from 100 to 400 dB/m: the cut-off values ​​we have chosen to indicate steatosis as absent, mild, moderate and severe were < 236 dB/m, ≥236 dB/m, ≥270 dB/m and ≥ 302 dB/m, respectively [20]. Finally, the NAFLD and FIB-4 fibrosis score were calculated for each patient, as previously reported [21, 22].

### Genetic studies

Genomic DNA was extracted from whole blood or 100 μL buffy coat, using a commercial kit (Invitrogen, Carlsbad, CA, USA), according to the manufacturer’s instructions. For each subject, 20 ng of genomic DNA was used for *PNPLA3* rs738409 allelic discrimination using TaqMan SNP Genotyping Assays (Life Technologies, Carlsbad, CA, USA) on the Applied Biosystems 7900HT Fast Real-Time PCR System (Life Technologies).

The DNA was then amplified by the polymerase chain reaction (PCR). 1 μL of DNA was added to 9 μL of the master PCR mix and specific primers were used to amplify the PNPLA3. The reaction protocol consisted of: i) a denaturation cycle at 95 °C for 3 min; ii) 35 cycles at 95 °C for 30 s, annealing at 62 °C for 30 s, extension at 72 °C for 30 s iii) extension at 72 °C for 10 min in a 96-well thermal cycler (Applied Biosystems 2720, ThermoFisher Scientific, Waltham, Massachusetts, United States). At the end of the reaction, the integrity of DNA extracted was assessed by gel electrophoresis. The bands were displayed under UV using the image system, interfaced with the Quantity One program. To define the genotype of the target genes, we performed a PCR of the restriction fragment length. We used NLA-III restriction enzymes for the PNPLA3 digest. All samples were amplified twice, discordant ones were performed a third time. Random samples were confirmed by direct genotyping, which provided concordant results in all cases; controls were included in all analyzed batches, and quality controls were used to verify the reproducibility of the results. Valid genotyping data were obtained for more than 99% of the analyzed subjects.

### Statistical analysis

Statistical data analysis was performed with the Stata statistical software package, version 13.1 (StataCorp LP, College Station, Texas, USA) and IBM SPSS® Statistics. The centrality and dispersion measurements of the data were either means, medians or interquartile intervals. Differences between control and prediabetics groups were assessed with Independent t-Test for continues variable and Mann-Whitney U test for ordinal variables. Paired Sample T test was utilized to analyze mean differences for continuous variables while Wilcoxon Signed-Rank Test was utilized to analyze mean differences for categorical variables. 1-wayANCOVA and Mann-Whitney U Test were run to determine the difference between the treatment with only Mediterranean diet versus silymarin plus Mediterranean diet after controlling for pre-intervention (baseline) biochemical parameters. To study strength and direction of association between categorical and continuous variables the nonparametric kendall’s (τb) tau-b correlation was run; this test was preferred over the Pearson chi-square test as the assumptions of either linearity or normality were not met. A chi-square test was used to study the association between the categorical variables PNPLA3 genotypes (CC or CG/GG) and LS (<7.9Kpa or > 7.9 Kpa); Phi (φ) and Cramer’s V measures were used to define the strength of the association.

The association of hypothetical predictors with hepatic rigidity was evaluated with a multiple linear regression model. A binomial logistic regression was also performed to ascertain the effects of gender, BMI, ALT, AST, GGT, HbA1c, cholesterol on the likelihood that participants have significant liver fibrosis defined as liver stiffness ≥7.9 kPa in prediabetic patients. Linearity of the continuous variables with respect to the logit of the dependent variable was assessed via the Box-Tidwell procedure. The significance level chosen for all statistical tests was 0.05 (two-tailed).

## Results

### Case-control study

#### Prevalence and main features of NAFLD in healthy controls group and in prediabetic patients prior to undergoing diet and/or herbal supplement administration

The demographic and clinical characteristics of the studied population are presented in Table 1. The median liver stiffness observed in the prediabetic group prior to any diet or herbal supplement administration was significantly higher than that observed in the healthy controls (6.8 [5.0–8.4] vs. 5.3 [4.3–6.0] kPa; *p* < 0.001). 4/126 (3%) of healthy controls had a LS compatible with significant fibrosis vs *n* = 61/212 prediabetic patients (29%) (*p* < 0.001). NAFLD fibrosis score and Fib-4 were also significantly increased in prediabetics patients. 49/212 patients (23%) in the pre-diabetic group versus 0% in control group had transaminases above the upper normal limit (p < 0.001). Also, Out of 49 patients with elevated transaminases 40 had LS > 7.9 kPa, while 9/49 patients had LS < 7.9 kPa. In the pre-diabetic group, a CAP value consistent with steatosis (ie, ≥236 dB/m) was detected in *n* = 123/212 patients (58%). 47 of these 123 prediabetic patients (38%) had a LS value not indicative of fibrosis (<5.9 kPa). Yet, of the 61 patients with significant fibrosis (LS > 7.9 KPa), 100% of them had a CAP value indicative of steatosis. In 89/212 of prediabetic patients without evidence of steatosis (42%), LS values indicative of mild fibrosis (between 5.9 and 7.9 kPa) were found. The statistical association between CAP values indicative of steatosis and LS values >5.9 kPa indicative of fibrosis was significant (*p* < 0.001).

**Table 1:** Main demographic and clinical characteristics of prediabetic subjects (n = 212) and healthy controls (n = 126) at baseline prior to intervention.

Variable	n = 212	n = 126	p value
Sex, Males/Females	157 / 56	94/ 32	0.96
Age, yr	61.4 ± 0.4	61.6 ± 0.4	0.77
Age at diagnosis of diabetes, yr	56 (52-58)	-	
Prediabetes duration, yr	6 (4-8)	-	
Body Mass Index, kg/m^2^	27.5 ± 0.2	27.2 ± 0.1	0.23
Waist circumference, cm	98.9 ± 0.9	99.5± 0.8	0.34
Females	94.8 ± 0.8	95.2 ± 1.0	0.45
Males	99.2 ± 0.7	99.0 ± 0.8	0.52
Steatosis grade (ultrasound)			
Absent	12 (5%)	20 (16%)	
Grade 1	100 (47%)	62 (49%)	
Grade 2	63 (29%)	34 (27%)	p=0.001
Grade 3	37 (17%)	10 (8%)	
Controlled attenuation parameter, dB/m	259 (239-271)	260 (240-277)	0.985
CAP ≥ 236 dB/m	123 (58)	65 (52)	0.52
Liver Stiffness, kPa	6.8 ± 1.9	5.3 ± 1.1	<0.001
< 5.9	79 (37%)	82 (65%)	p<0.001
> 5.9-7.9	72 (34%)	40 (32%)	
> 7.9	61 (29%)	4 (3%)	
Total cholesterol, mg/dl	168.1 ± 0.9	171 ± 1.3	0.074
HDL Cholesterol, mg/dl	47.6 ± 0.6	48.1 ± 0.7	0.21
Triglycerides, mg/dl	139.8 ± 1.65	127.6 ± 2.7	<0.001
Aspartate aminotransferase, IU/L	28.6 ± 0.6	25.26 ± 0.6	<0.001
Alanine aminotransferase, IU/L	38.8 ± 0.4	23.2 ± 0.7	<0.001
AST, ALT, IU/L >Upper Normal Limit	49 (23%)	0 (0%)	<0.001
Gamma glutamyltranspeptidase, IU/L	31.5 ± 0.8	31.6 ± 0.6	0.88
Platelet count, ×10^9^/L	248.6± 1.3	264.4 ± 2.4	<0.001
Fasting glucose, mg/dl	110.8 ± 0.4	90.98 ± 1.1	<0.001
Albumin, g/L	38.5 ± 0.1	40.7 ± 0.2	<0.001
HbA1c, mmol/mol	43.3 ± 0.2	30.3 ± 0.3	<0.001
NAFLD Score, unit	-0.49 ± 0.05	-0.249 ± 0.06	0.001
Significant fibrosis unlikely	40 (19%)	36 (2%)	
Indeterminate	158 (74%)	87 (69%)	0.014
Significant fibrosis likely	14 (7%)	3 (2%)	
FIB-4, unit	1.11 ± 0.02	0.60 ± 0.02	<0.001
Significant fibrosis unlikely	135 (64%)	122 (97%)	
Indeterminate	77 (36%)	4 (3%)	<0.001
Significant fibrosis likely	0 (0)	0 (0)	

Differences between groups was assessed with Independent t-Test for continuous variable and Mann-Whitney U test for ordinal variables (steatosis grade, liver stiffness classes, NAFLD score and FIB-4)

Abbreviations: CAP controlled attenuation parameter, HDL-C high density lipoprotein cholesterol, HbA1c glycosylated hemoglobin; NAFLD nonalcoholic fatty liver disease, FIB-4 fibrosis-4

^a^Data presented as number (%) for ordinal variables or means ± s.e for continuous variables

**Table 2:** Nonparametric kendall’s (τ_b_) tau-b correlation between patatin like phospholipase domain containing 3 (PNPLA3) genotypes (CC or CG/GG) and biochemical parameters.

	Patatin like phospholipase domain containing 3 (PNPLA3)			
	CC (n=100)	CC/GG (n=112)		
continuous variables	mean ± std deviation	mean ± std deviation	τ_b_ correlation coefficient	Sig. (2-tailed)
BMI	25.9 ± 1.03	27.851 ± 1.52	0.524	<0.001
ATL	25.2 ± 5.79	34.94 ± 7.23	0.545	<0.001
AST	22.83 ± 4.61	33.07 ± 8.05	0.523	<0.001
GGT	27.16 ± 8.00	39.3 ± 0.80	0.553	<0.001
HbA 1c	43.36 ± 2.34	44.48 ± 2.41	0.196	0.001
cholesterol	159.28 ± 7.49	174.13 ± 11.1	0.552	<0.001
triglycerides	101.88 ± 10.41	133.87 ± 24.28	0.547	<0.001

The nonparametric kendall’s (τ_b_) tau-b correlation: strength and direction of the association that exists between patatin like phospholipase domain containing 3 (PNPLA3) genotypes (CC or CG/GG) and BMI, AST, ALT, HbA1c, GGT, cholesterol and triglycerides

CC genotype was coded as “0” while CG/GG genotypes were coded as “1”. τ_b_ values range from -1 to +1

**Table 3:** A chi-square test for association was conducted between for PNPLA3 genotypes (CC or CG/GG) and LS (<7.9Kpa or >7.9 Kpa).

PNPLA3 genotypes (CC or CG/GG) * LS (<7.9KPa or >Kpa) Crosstabulation					
			LS (Kpa)		Total
			<7.9	≥7.9	
	CC	Count (n)	100	0	100
		Expected Count	71.2	28.8	100
		% within CC	100.00%	0.00%	100.00%
	CG/GG	Count (n)	51	61	112
		Expected Count	79.8	32.2	112
		% within CG/GG	45.50%	54.50%	100.00%
Total		Count (n)	151	61	212
		Expected Count	151	61	212
		% within CC or CG/GG	71.20%	28.80%	100.00%
Chi-Square Test					
	Value	df	Asymptotic Significance (2-sided)		
Person Chi-Square	76.466	1	<0.001		

χ2(1) = 76.466, p <0.001. φ = 0.601, p <0.001

#### Factors associated with significant fibrosis

Table 4 presents the univariate analysis of demographic and clinical factors associated with significant fibrosis in prediabetic state, defined as LS ≥7.9 kPa. BMI is the strongest predictor of a stiffness that suggests significant fibrosis, while age of onset and duration of diabetes do not have appreciable effects. Elevated transaminases are significantly associated with liver stiffness values >7.9 kPa (*p* < 0.001) as 49/55 patients with LS > 7.9 have elevated transaminases. Yet, 0/72 patients with LS between 5.9 and 7.9 kPa have elevated transaminases. There is no significant correlation between the levels of LS and the categories of significant fibrosis predicted by the NAFLD fibrosis score and Fib-4 (*p* = 0.058 and *p* = 0.063 respectively).

**Table 4:** Univariate analysis of factors associated with significant liver fibrosis defined as liver stiffness ≥7.9 kPa in prediabetic patients at baseline prior to intervention.

Variable	LS <7.9 kPa (n = 151)	LS≥7.9 kPa (n = 61)	P value
Male gender	112 (74)	45 (74)	0.07
Age, yr	60 (54-65)	64 (61-65)	0.088
Age at diagnosis of prediabetes, yr	55 (50-56)	56 (52-59)	0.098
Prediabetes duration, yr	5 (3-6)	6 (3-7)	0.145
Body mass index, kg/m^2^	26 (25.0-27.9)	28.8 (28.0-29.0)	0.014
Waist circumference, cm	98 (94-99)	104 (102-105)	0.042
Female	93 (90-95)	99 (97-99.5)	0.021
Males	99 (95-99)	101 (100-103)	0.032
Steatosis grade (ultrasound)			
Absent	10 (7)	2 (3)	
Grade 1	80 (53)	20 (33)	
Grade 2	41 (27)	22 (36)	
Grade 3	20 (13)	17 (28)	0.002
CAP, dB/m	249 (231-260)	280 (275-282)	0.040
Total cholesterol, mg/dL	168 (159-174)	167 (149-179)	0.807
HDL cholesterol, mg/dL	44 (40-47)	49 (39-50)	0.295
Triglycerides, mg/dL	120 (112-145)	160 (143-169)	0.055
Aspartate aminotransferase, IU/L	23 (19-27)	37 (35-41)	0.003
>Upper Normal Limit	4 (3)	12(19)	<0.001
Alanine aminotransferase, IU/L	24 (21-32)	37 (35-41)	<0.014
>Upper Normal Limit	14 (9)	19 (31)	<0.001
Gamma glutamyltranspeptidase, IU/L	28 (21-37)	40 (37-41)	0.097
>Upper Normal Limit	20 (9)	17 (18)	0.020
Platelet count, ×10^9^/L	255 (244-259)	250 (244-254)	0.501
Fasting glucose, mg/dL	108 (102-117)	115 (109-117)	0.125
Albumin, g/L	39 (37.0-39.0)	37.9 (37.2-38.2)	0.463
HbA1c, mmol/mol	43 (40-44)	44 (41-47)	0.064
NAFLD Score, unit	-0.70 (-0.90-0.50)	-0.210 (-0.50-0.30)	0.058
FIB-4, unit	0.91 (0.76-0.99)	1.62 (1.345-1.755)	0.063

Abbreviations: CAP controlled attenuation parameter, HDL high density lipoproteins, HbA1c glycated hemoglobin, NAFLD nonalcoholic fatty liver disease, FIB-4 fibrosis-4

^a^For continuous variables, the measures of central tendency and dispersion are medians and interquartile range (IQR), respectively. ^b^Categorical variables are shown as frequencies (%)

Furthermore, a binomial logistic regression was performed to ascertain the effects of gender, BMI, ALT, AST, GGT, HbA1c, cholesterol on the likelihood that participants have significant liver fibrosis defined as liver stiffness ≥7.9 kPa in prediabetic patients (Table 5). The Hosmer and Lemeshow test is not statistically significant (*p* = 0.76;), indicating that the model is a good fit. The logistic regression model was statistically significant, χ2(7) = 191.9, *p* < .001. The model explained 85.2% (Nagelkerke R2) of the variance in significant fibrosis (≥7.9 kPa). A seven-predictor logistic model was generated regarding the relationship between the likelihood that a patient develops significant liver fibrosis and his gender, BMI, ALT, AST, GGT, HbA1c, cholesterol. Predicted logit of (Significant liver fibrosis) = −71.821 + (−0.126) *gender + (2.084)* BMI + (0.254)*AST + (0.183)*ALT+ (−0.045)*HbA1c + (0.121)*GGT + (−0.029)* cholesterol. Female gender is coded as 0 and male gender as 1. Of the predictor variables three were statistically significant: BMI, AST, and ALT with *p* value of <0.001, 0.006, 0.04 respectively (Table 5). Females and males had similar odds of having significant fibrosis.

**Table 5:** Binomial logistic regression: prediction of liver fibrosis (aka stiffness ≥7.9 kPa) based on gender, BMI, ALT, AST, GGT, HbA1c, cholesterol.

Variables in the Equation	B	S.E.	Wald	df	Sig.	Exp(B)	95% C.I. for EXP(B)	
							Lower	Upper
GENDER (F=0, M=1)	-0.126	0.747	0.028	1	0.866	0.882	0.204	3.814
BMI	2.084	0.499	17.454	1	0.00003	8.035	3.023	21.357
AST	0.254	0.092	7.597	1	0.006	1.29	1.076	1.545
ALT	0.183	0.089	4.219	1	0.04	1.201	1.008	1.429
HBA1c	-0.045	0.131	0.118	1	0.731	0.956	0.739	1.236
GGT	0.121	0.123	0.966	1	0.326	1.129	0.886	1.437
COLESTROLOL	-0.029	0.022	1.801	1	0.18	0.972	0.931	1.013
Constant	-71.821	17.176	17.484	1	0	0		

Exp (B) = odds ratio; χ2(7) = 191.9, p < .001; Nagelkerke R^2^ = 85.2%; Hosmer and Lemeshow test: p = 0.76; Predicted logit of (Significant liver fibrosis) = -71.821 + (− 0.126)*gender + (2.084)* BMI + (0.254)*AST + (0.183)*ALT+ (-0.045)*HbA1c + (0.121)*GGT + (-0.029)* cholesterol. Female gender is coded as 0 and male gender as 1

#### Influence of genetic factors on fibrosis progression

As for rs738409 (PNPLA3), 47.2% of patients were C/C (wild type) homozygotes, while 52.8% had at least one G (variant) allele. As shown in Table 3, there is a statistically significant association between PNPLA3 genotypes (CC or CG/GG) and LS (<7.9 kPa or > 7.9 kPa), χ2(1) = 76.466, *p* < 0.001. The association between PNPLA3 genotypes CG/GG and LS ≥ 7.9 was strong, φ = 0.601, p < 0.001. Furthermore, having classified the patients into two groups, those with LS suggesting significant fibrosis (≥ 7.9 kPa) and those without significant fibrosis (<7.9 kPa), 54.5% of the group with either CG or GG genotypes had significant fibrosis while 0% of the CC genotype group had significant fibrosis. This difference was analyzed with the test of two proportions also known as the chi-square test of homogeneity; the difference in proportions is of 0.54, *p* < .001.

#### Interaction between patatin like phospholipase domain containing 3 (PNPLA3) genotypes (CC or CG/GG), BMI and biochemical parameters

The nonparametric kendall’s (τb) tau-b correlation was run to determine the measure of the strength and direction of the association that exists between patatin like phospholipase domain containing 3 (PNPLA3) genotypes (CC or CG/GG) and BMI, AST, ALT, HbA1c, GGT, cholesterol and triglycerides. Each biochemical parameter and CG/GG genotype had a positive correlation coefficient τb; each association was statistically significant (*p* < 0.001 or *p* = 0.001 for HbA1c), and the strength of the associations was moderate/strong for each variable (Table 2).

Finally, a multiple regression model was built to predict LS in relation to BMI to PNPLA3 genotype. LS provided for patients was 1.023 + 0.028 (BMI in kg/m2) + 0.078 (PNPLA3 genotype, coded 0, 1 or 2 based on the number of G alleles present). Based on this model, the predicted value of liver stiffness can range from a normal for patients with BMI < 28 who are rs738409 C/C homozygous, to one suggestive of significant disease progression in patients with BMI > 28 who are homozygous rs738409 G/G.

Of the 22 patients who are homozygous rs738409 G/G, 13 of them had LS > 7.9 KPa and of those 13 patients all of them had BMI > 28. 9/22 patients homozygous for rs738409 G/G had LS < 7.9 KPa and of those 9 patients only 2 of them (22%) had a BMI >28.

### Intervention study

#### Effects of treatment on regression of hepatic and diabetic diseases

Pre-diabetic patients, who undergo a Mediterranean diet with weight reduction greater than 5% of the original body weight either alone or in conjunction with nutraceutical, experience a significant regression of fibrosis and a significant decrease in ALT, HbA1c, FBS after 6 months (*p* < 0.001 for each variable, Table 6). Prediabetic patients who followed a Mediterranean diet alone (group 1) experienced a significant regression of fibrosis after 6 months (mean LS is 6.70 vs. 5.90); Mediterranean diet in conjunction with silymarin treatment (group 2) also leads to regression of fibrosis (mean LS is 5.40 vs. 6.80), yet there is no significant difference when compared to diet alone (*p* = 0.696; Table 6). Similarly, there was no significant difference between the two groups after treatment in ALT, AST and FBS ( Table 6). Interestingly, diet with weight reduction greater than 5% together with silymarin regimen (group 2) leads to a statistically significant reduction in HbA1c when compared to diet alone (group 1) (95% CI: 37.8–38.6 vs 39.5–40.3, *p* < 0.001, Table 6).

**Table 6:** Mean differences of biochemical parameters within each group and between groups 6 months after either only diet or regimen of diet plus silymarin (group 1, n = 104; group 2, n = 108).

Variable	Group 1			Group 2			Mean Difference between group 1 and group 2 after 6 mo; F or Z score, p value
	Baseline Mean ± s.e., 95% CI	After 6 mo mean ± s.e., 95% CI	Paired differences, t or z scores	Baseline Mean ± s.e. 95% CI	After 6 mo mean ± s.e., 95% CI	Paired differences, t or z scores	
BMI ( kg/m^2^)	26.65 ± 0.13 (26.4 – 26.9)	24.61 ± 0.21 (24.2 - 25.0)	2.04 ±1.5, t(103) = 14.1 p < .001	26.88± 0.14 (26.60-27.16)	25.12 ± 0.1 (24.9 -25.3)	1.73 ±1.7, t(107) = 10.6 p < .001	0.7, 0.25
Steatosis grade							
Absent	6 (6)	14 (11)	Z = 2.376 p =0.017	6 (6)	12 (11)	Z = 2.705, p =0.007	0.435, 0.662
Grade 1	50 (48)	60 (59)		50 (46)	63 (59)		
Grade 2	30 (29)	20 (20)		33 (31)	22 (20)		
Grade 3	18 (17)	10 (10)		19 (17)	11 (10)		
CAP, dB/m	253.16± 1.87 (249.5-256.9)	246.6 ± 1.75 (243.1-250.1)	5.98 ±18.75 t(103) = 3.2 p 0.002	254.72±1.92 (250.9– 258.5)	244.8 ±1.72 (241.39 – 248.17)	9.3 ±18.1, t(107) = 5.4 p < .001	0.568, 0.452
Liver Stiffness <7.9 kPa ≥7.9 kPa	6.84 ± .18 77 (71) 30 (29)	5.58 ± .03 90 (86) 18 (17)	1.29 ±0.74 t (103) = 17.9 p < .001	6.9 ± .17 77 (71) 31 (29)	5.57 ± 0.03 93 (86) 15 (14)	1.31 ±0.75, t (107) = 18.4 p < .001	0.153, 0.696
AST, IU/L	27.80± 0.85 (26.1-29.5)	27.8± 0.4 (27.0-28.6)	0.09 ± 4.9 t(103) = 0.194 p = .871	28.07± .84 (26.4-29.7)	27.5-0.4 (26.8-28.3)	0.438 ± 5.54, t (107) = 0.836 p = .404	0.163, 0.687
ALT, IUL	29.8 ± 0.8 (28.2-31.4)	29.2 ± 0.3 (28.7-29.8)	0.68 ± 3.1 t (103) = 2.22 p = .028	30.13 ± 0.8 (28.5-31.7)	29.3 ± 0.3 (28.8-29.8)	0.78 ± 3.29, t(107) = 2.53 p = .013	0.01, 0.921
Fasting glucose, mg/dL	117.9 ± .38 (117.1-118.7)	99.4 ± .37 (98.6-100.1)	18.5 ±5.21 t(103) = 36.7 p < .001	117.91 ± .37 (117.2-118.1)	99.41 ± .36 (98.7-100.1)	18.54 ±5.16, t(107) = 38.01 p < .001	0.003, 0.955
HbA1c, mmol/mol	44.12 ± .23 (39.6 – 40.6)	39.9 ± .2 (39.5 – 40.3)	4.26 ± 2.91 t(103) = 15.2 p < .001	44.0 ± .25 (43.5-44.5)	38.1 ± .2 (37.8-38.6)	5.78 ±2.78, t(107) = 21.99 p < .001	35.31, <0.001
NAFLD Score, unit	-0.33 ± .06 (-0.45 -0.21)	-0.65 ± .08 (-0.81- 0.491)	0.28 ± 0.98 t(103) = 15.2 p = .004	-0.34± .05 (-0.24- -0.44)	-0.68 ± .08 (-0.83 - 0.52)	0.35 ±0.98, t(107) = 3.73 p < .001	0.054, 0.817
FIB-4, unit	1.17± .04 (1.09-1.25)	0.92± .01 (0.89-0.94)	0.26 ± 0.19, t(103) = 14.15 p < .001	1.19 ± .04 (1.10 – 1.27)	0.91 ± .01 (0.89 - 0.94)	0.27 ±0.19, t(107) = 14.68 p < .001	0.44, 0.83

Abbreviations: CAP controlled attenuation parameter, NAFLD nonalcoholic fatty liver disease, FIB-4 fibrosis-4

^a^For continuous variables, the measures of central tendency are means. ^b^Categorical variables are shown as frequencies (%). Paired Sample T test was used to analyze mean paired differences for continues variables while Wilcoxon Signed-Rank Test was used for categorical variables. For mean difference across different treatment groups at the end of 6 months, ANCOVA statistics (F score) was used for continuous dependent variables while Mann-Whitney U Test (Z score) was used for ordinal dependent variables

#### Safety

In the present study, oral administration of 210 mg silymarin every 12 h for 6 months did not produce any adverse reactions. No abnormalities were noted either on physical exam or on the chemistry panel. Specifically, no dyspepsia, diarrhea or headache and dizziness have been reported. Conformity and tolerability have been described as “optimal”.

## Discussion

NAFLD is a slowly progressive disease, where the rate of progression is influenced by the stage at initial diagnosis, as observed in patients with non-alcoholic steatohepatitis [23]. The prevalence of NAFLD is higher among diabetics than in the general population [24]. Furthermore, patients with prediabetes are more likely to have more severe histological forms of NAFLD [25]. In patients with prediabetes and type 2 diabetes, liver lipogenesis is elevated while fatty acid oxidation remains decreased; at the same time, peripheral insulin resistance increases fatty acid release from adipose tissue and promotes their hepatic uptake. This milieu favors the progressive development of NAFLD in patients with prediabetes, and increases the likelihood of NAFLD to progress to NASH and cirrhosis [26, 27]. These results support the idea that in prediabetic patients, early diagnosis and treatment of NAFLD is paramount.

Our study shows that in an unselected cohort of patients with prediabetes, not previously known to have NAFLD, the prevalence of liver disease is unexpectedly high. The clinical characteristics of the recruited patients explain most of the variability observed, with a significant contribution offered by the genetic profile. As shown in Table 4, the age and the duration of the prediabetic condition do not contribute significantly to the prevalence of NAFLD. Yet, as previously shown, we also observe that excess body weight facilitates progression of liver fibrosis (Tables 4, 5) [28].

The reason obesity affects the pathophysiology of liver damage is likely related to the fact that leptin induces pro-inflammatory and angiogenic cytokines, thus altering the liver wound-healing response [29–31].

In recent years, in addition to environmental factors and comorbidities, genetic predictors of progression of liver disease have also been identified [32]. The strongest genetic predictor of progression of chronic liver disease is SNS rs738409 in the PNLPA3 gene, which encodes a membrane-bound triacylglycerol lipase mediating the hydrolysis of the triacylglycerol. This “missense” mutation causes a reduction in the enzymatic activity of PNPLA3, leading to the development of macrovascular steatosis [33, 34]. In our study, we evaluate how the PNPLA3 mutation plays a part in the progression of liver disease in prediabetes. Increased risk of hepatic fibrosis in patients with DMT2 who are carriers for the mutated variant of rs738409 was previously reported [35, 36]; yet, the estimate of liver fibrosis was based on Fibrotest rather than on liver stiffness. Herein, we show the existence of significantly increased liver stiffness in mutated heterozygous and homozygotes compared to wild-type PNPLA3 in prediabetic patients (Table 3). We can thus infer that in prediabetic patients, PNLPA3 contributes to the progression of liver disease; nevertheless, in prediabetics, unlike in the general population, the net effect may be driven by excess BMI. In fact, our results indicate that of the 22 patients who are homozygous rs738409 G/G, 13 of them had LS > 7.9 kPa and of those 13 patients all of them had BMI > 28. Instead, in the 9 patients homozygous for rs738409 G/G who had LS < 7.9 kPa only 2 of them (22%) had a BMI > 28. This observation suggests that elevated BMI index may synergistically modulate fibrosis progression in specific variants of PNLPA3. Similarly, increase in BMI augments the effect of M variant on the prevalence of cirrhosis [37]. Therefore, a future study should be conducted on patients with BMI greater >30 to further analyze the significance of this interaction between PNPLA3 gene and BMI in the contest of prediabetes and progression of NAFLD to significant fibrosis.

Though genetic variants may be considered in the construction of prediction models aimed to detect the progression of NAFLD to fibrosis in patients with prediabetes, the use of Fibroscan in this patient population remains paramount. The prevalence of moderate to advanced steatosis scored using CAP, independently of BMI, does not significantly differ between the control group and patients with prediabetes (see Table 1, 52% vs 58%, *p* = 0.3); nevertheless, when using LS as a measure of liver fibrosis, there appears to be a significant difference in advanced steatosis when comparing these two groups (Table 1, 3% vs 29%, *p* < 0.001), thus indicating that prediabetic state plays a critical factor in the progression of steatosis to fibrosis. The role of TE should be particularly underscored in that group of prediabetic patients with mild fibrosis (LS 5.9–7.9 kPa) as transaminase levels are not elevated in this patient group (see Table 4); this indicates that transaminases levels are not a good indicator of mild fibrosis and can lead to a sense of false reassurance in this group of patients. Nonetheless, we show that transaminases, together with BMI, remain the principle indicators associated with significant fibrosis (Tables 4 and 5).

As aforementioned, lifestyle modifications are recommended for control of NAFLD [11, 12]. It has been demonstrated that an intensive weight loss program led to resolution of NASH and improvement of fibrosis [38]. Moreover, follow-up studies on morbidly obese patients that underwent bariatric surgery have also showed that the extent of weight loss correlates with the degree of NASH and fibrosis [39]. We herein show that prediabetic patients who follow a Mediterranean diet and achieve weight loss greater than 5% experienced a significant regression of fibrosis after 6 months (mean LS is 6.70 vs. 5.90; *p* < 0.001, Table 6). In recent years, the use of natural products has been considered for the treatment of hyperglycemia. Furthermore, the protective effects of silymarin as a powerful ROS scavenger, and its hepato-protective effects via attenuation of pro-inflammatory gene expression in the liver have been discussed previously [40]. There is also recent experimental evidence that in diabetic rats treated with silymarin and in which weight reduction was achieved, markers of hepatic injury were significantly reduced and favorable histopathological changes were showed [41]. Randomized control trials have shown that regimens with either silymarin alone or its most active biological compound silybin lead to liver aminotransferase reduction in patients with NAFLD after only 3 months [42–44]. One of these studies also investigated the role of silymarin therapy in the context of PNPLA3 polymorphism and showed that PNPLA3 G-allele carriers did not receive any benefits from treatment with silymarin. Furthermore, silymarin together with lifestyle modification has an increased safety profile relative to other anti-oxidant inhibitors or anti-fibrotic agents while conserving liver-enzyme ameliorating effects in patients with NAFLD [45, 46]. A systematic review of clinical trials conducted between 2003 and 2008 did not report any significant side effects attributable to silymarin therapy compared to the placebo-treated population and also did not show any significant interactions with other drugs [47]. Given its safety profile, silymarin continues to be widely used in the treatment of acute or chronic hepatitis. Yet, no study has shown regression of liver fibrosis with silymarin therapy upon assessment with Fibroscan. We thus investigated whether in patients with hepatic steato-fibrosis and prediabetes the reduction of body weight > 5% and the intake of silymarin reduces progression of comorbid liver disease. We show that Mediterranean diet either alone or in conjunction with silymarin regimen leads to a significant reduction of liver fibrosis, normalization of ALT and significant reduction in HbA1c and FBS. Nevertheless, after controlling for pre-intervention (baseline) biochemical parameters, there is no difference in fibrosis regression between the treatment with only Mediterranean diet versus silymarin plus Mediterranean diet (*p* = 0.696). Finally, it should be underscored that both Mediterranean diet with weight reduction greater than 5% alone and in conjunction with silymarin treatment lead to significant reduction of HbA1c (*p* < 0.001, Table 6). Interestingly, diet with weight reduction greater than 5% together with silymarin regimen leads to a statistically significant reduction in HbA1c when compared to diet alone (95% CI: 37.8–38.6 vs 39.5–40.3, p < 0.001, Table 6). Interestingly, Li et al. showed the role of silybin in the prevention of palmitate-induced insulin resistance and inhibition of the IRS-1/PI3K/Akt pathway in skeletal muscle cells [48]. These observations imply that silymarin may play an important role in glycemic control and it may be of interest to investigate its anti-glycemic properties in isolation in future studies. Among the various limitations of this study, we must note the fact that Fibroscan is not the best method to characterize NAFLD. However, compared to the magnetic resonance evaluation, the evaluation with TE is much more practicable and at lower cost in clinical practice.

In conclusion, it is critical to acknowledge that patients with prediabetic status have a high prevalence of liver disease characterized by significant fibrosis, which goes largely unnoticed in the absence of symptoms and laboratory abnormalities. We herein show how increased BMI and genetic factors represent critical variables in the risk stratification analysis of prediabetic patients with NAFLD. Lastly, we propose that Mediterranean diet together with intake of the nutraceutical silymarin contributes to the regression of liver damage and diabetes markers.
